# Rapid Unconscious Acquisition of Conditioned Fear with Low-Spatial-Frequency but Emotionally Neutral Stimuli

**DOI:** 10.34133/research.0181

**Published:** 2023-06-27

**Authors:** Yujie Chen, Si Chen, Zhongju Sun, Xilei Zhang, Xiangyong Yuan, Liang Wang, Yi Jiang

**Affiliations:** ^1^ State Key Laboratory of Brain and Cognitive Sciences, CAS Center for Excellence in Brain Science and Intelligence Technology, Institute of Psychology, Chinese Academy of Sciences, Beijing 100101, China.; ^2^ Department of Psychology, University of Chinese Academy of Sciences, Beijing 100049, China.; ^3^ Chinese Institute for Brain Research, Beijing 102206, China.; ^4^ CAS Key Laboratory of Mental Health, Institute of Psychology, Chinese Academy of Sciences, Beijing 100101, China.

## Abstract

It has long been proposed that emotionally “prepared” (i.e., fear-related) stimuli are privileged in the unconscious acquisition of conditioned fear. However, as fear processing is suggested to highly depend on the coarse, low-spatial-frequency (LSF) components of the fear-related stimuli, it is plausible that LSF may play a unique role in the unconscious fear conditioning even with emotionally neutral stimuli. Here, we provided empirical evidence that, following classical fear conditioning, an invisible, emotionally neutral conditioned stimulus (CS+) with LSF, but not with high spatial frequency (HSF), can rapidly elicit stronger skin conductance responses (SCRs) and larger pupil diameters than its CS− counterpart. In comparison, consciously perceived emotionally neutral CS+ with LSF and HSF elicited comparable SCRs. Taken together, these results support that the unconscious fear conditioning does not necessarily entail emotionally prepared stimuli but prioritizes LSF information processing and highlight the crucial distinctions between the unconscious and the conscious fear learning. These findings not only coincide with the postulation that a rapid, spatial-frequency-dependent subcortical route is engaged in unconscious fear processing but also suggest the existence of multiple routes for conscious fear processing.

## Introduction

To survive in complex environments, humans have evolved to automatically learn about what stimuli potentially predict threats and then exhibit learned fear responses to these stimuli even when they were masked and therefore rendered invisible [1]. Decades ago, through investigating such forms of fear learning, researchers have revealed that the emotionally “prepared” [2] stimuli, such as snakes, spiders, or angry faces that signal potential dangers, are privileged in the unconscious acquisition of the conditioned fear than the emotionally neutral stimuli, such as mushrooms or flowers [3,4]. In other words, participants are able to learn the association between the masked fear-relevant stimuli (i.e., conditioned stimuli [CSs]) and the aversive stimuli (i.e., unconditioned stimuli, [USs]), whereas this fear learning cannot be acquired when the masked CSs are replaced by fear-irrelevant ones [3,4]. This opinion constitutes the core of the prepared theory [1,2]. It underlines that fear-relevant stimuli that threaten our survival over the long evolutionary history have been endowed with an innate propensity to acquire associations with negative events.

Given the prevalence of this theory, some following studies have implicitly accepted the preparedness explanation and preferentially chosen the threat-related stimuli to investigate the unconscious fear conditioning [5–8]. However, the preparedness theory is still controversial, and whether the unconscious fear conditioning necessarily entails fear-relevant stimuli is under debate [9,10]. One study has found that a patient with completely cortical blindness was able to acquire the association between an unseen simple visual cue and an aversive electric shock [11]. Some other studies showed that a pretrained association between masked neutral faces (or even simple geometric shapes) and electric shocks can facilitate a later relearning of the same CS–US relationship, demonstrating unconscious fear conditioning in neurotypical participants [12,13]. Although these findings that failed to support the specificity of fear-relevant CSs in the unconscious fear conditioning pose great challenges to the preparedness theory, the evidence so far is insufficient to illustrate why the unconscious fear conditioning should or should not depend on the emotional “prepared” stimuli.

A critical but rarely explored factor in the unconscious fear conditioning is the spatial frequency information of the CSs, in which low-spatial-frequency (LSF) information corresponds to the coarse features whereas high-spatial-frequency (HSF) information depicts the fine details [14]. A line of studies have examined the behavioral and neural relationship between spatial frequency and emotional response, and some of them converged to propose that the processing of threat-related stimuli might predominantly rely on the coarse LSF components ([15–18], see also the debates on this postulation [14,19]). Particularly, they highlighted that the LSF features per se can suffice to activate the subcortical structures involved in the processing of emotional information [15–17], even when the stimuli are rendered invisible [18,20]. For instance, functional magnetic resonance imaging and intracranial electrophysiological findings from both healthy participants and a blindsight patient showed that the fearful stimuli with the LSF components evoked rapid and stronger activation in the amygdala [15,16,20] and other subcortical structures, such as the superior colliculus and thalamus, as compared with stimuli with only the HSF components [17].

**Fig. 1.**
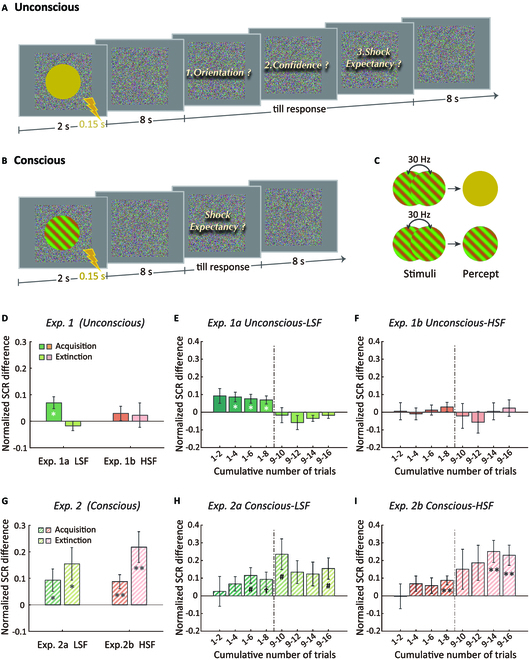
Procedure, stimuli, and results of Experiments 1 and 2. An exemplar CS+US trial in (A) Experiment 1a (unconscious fear conditioning) and (B) Experiment 1b (conscious fear conditioning). (C) CSs of LSF. Top row: invisible CSs generated by rapidly counterphase flickering at 30 Hz; bottom row: visible CSs generated by rapidly flickering at the same phase. The normalized SCR differences between CS+ and CS− for LSF and HSF in Experiment 1 (D) and Experiment 2 (G), respectively. The accumulation of the normalized SCR differences across trials for the unconscious LSF and HSF conditions in Experiment 1 were separately illustrated in (E) and (F), while the accumulation of the normalized SCR differences across trials for the conscious LSF and HSF conditions in Experiment 2 were illustrated in (H) and (I), respectively. The x-axis indicated that the normalized SCR differences were accumulated at a pace of 2 trials for the acquisition and extinction stages, respectively (i.e., acquisition: 1–2, 1–4, 1–6, 1–8; extinction: 9–10, 9–12, 9–14, 9–16). The dot-dash lines divided the acquisition and the extinction stages. Each error bar represents the standard error of the mean. False discovery rate corrected: †*P* = 0.076, #*P* = 0.058, *P < 0.05, **P < 0.01.

**Fig. 2.**
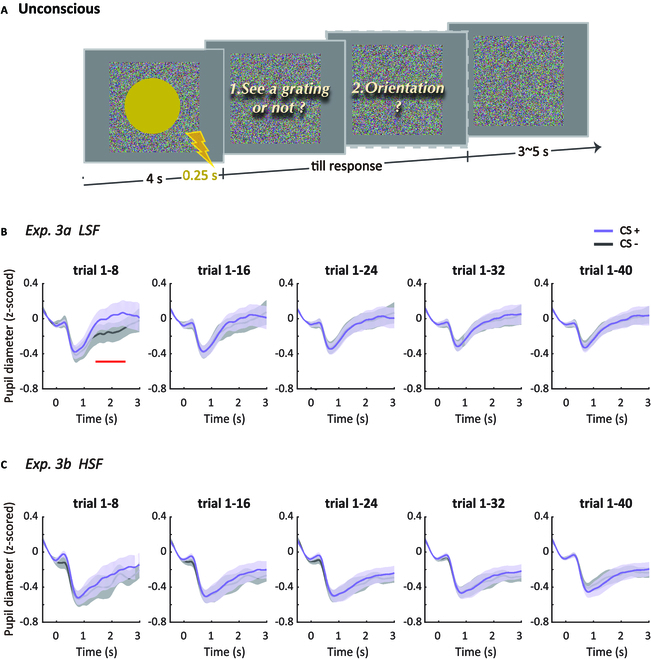
Procedure and results of Experiment 3. (A) The procedure of a CS+US trial. Participants answered the second question only if they reported that they saw the grating in the yellow disk. The cumulatively averaged pupil diameter in response to CS+ and CS− at a pace of 8 trials in the LSF (B) and HSF (C) conditions, respectively. The red line denotes the time points when there were significant pupil diameter differences between CS+ and CS− (corrected by cluster-based permutation).

Given the aforementioned evidence, it is plausible that the coarse features (i.e., the LSF components) embedded in the visual stimuli are critical for the processing of fear-related information. This raises a possibility that the spatial frequency may also play an important role in the successful unconscious acquisition of the conditioned fear. In other words, the unconscious fear conditioning may not essentially entail emotionally prepared stimuli but depends on the LSF components. To test this alternative hypothesis, the current study investigated the unconscious fear conditioning processes using emotionally neutral stimuli with different spatial frequencies (i.e., LSF or HSF). We chose chromatic gratings either with LSF or HSF as CSs and manipulated their visibility to compare the fear conditioning processes under different consciousness states [18] (Fig. 1A and B and Fig 2A). The CSs differed in their orientations (leftwards or rightwards) and were rendered invisible through a Critical Flicker-Fusion Frequency (CFF) paradigm (Fig. 1C), in which the CSs with counterphase chromatic gratings alternating at a frequency of 30 Hz would be perceptually fused into a unified color patch such that their orientations were completely invisible [21,22]. Compared with the indirect measures of participants’ awareness of CS–US contingencies [23] or the brief perceptual interference of backward masking [12,13], the CFF paradigm can effectively ensure that participants are not aware of these CSs for a relatively long duration. Moreover, we adopted 2 classical physiological measurements which have been widely employed in fear conditioning research, namely, the skin conductance responses (SCRs) and the pupillary responses [24]. The results from both measurements together revealed that the unconscious acquisition of conditioned fear can take place rapidly with LSF but emotionally neutral stimuli.

## Results

### Emotionally neutral stimuli with LSF but not HSF rapidly elicit SCRs during unconscious fear conditioning

Experiment 1 examined whether the unconscious fear conditioning can be established for invisible emotionally neutral stimuli with LSF or HSF components. In Experiment 1a, both the CS+ and CS− were of LSF (1 cycle per degree), while in Experiment 1b, both the CS+ and CS− were of HSF (9 cycles per degree). The main fear conditioning experiment consisted of 2 stages. In the fear acquisition stage, one of the 2 chromatic gratings (i.e., CS+) was repeatedly paired with aversive electric shocks (i.e., US), whereas in the extinction stage, the CS+ was presented alone without being paired with the US. The participants in the experiments were strictly screened to ensure that they were not aware of the orientations of the CS+ and CS− and also performed at chance level when being forced to judge the orientations (formatted in M ± SD, Experiment 1a: 0.48 ± 0.08, *t*(27) = −1.28, *P* = 0.211, Cohen’s *d* = 0.24; Experiment 1b: 0.51 ± 0.05, *t*(27) = 1.09, *P* = 0.285, Cohen’s *d* = 0.21; see Materials and Methods for the inclusion criteria of participants).

To control for the intersubject variability, the SCR induced by the CS+ and CS− were normalized and converted to the SCR difference using an algorithm, $\frac{\mathrm{CS}+-\mathrm{CS}-}{\mathrm{CS}++\mathrm{CS}-}$ [8]. The result of Experiment 1a revealed that the normalized SCR difference in the acquisition stage was significantly larger than zero (*t*(27) = 2.99, *P* = 0.024, Cohen’s *d* = 0.57), which denoted that the invisible CS+ with LSF components elicited significantly larger SCR than the invisible CS− counterpart (Fig. 1D, left). The normalized SCR difference in the extinction stage did not significantly deviate from zero (*t*(27) = −0.98, *P* = 0.447, Cohen’s *d* = 0.19). Using a cumulative analysis, we further demonstrated that the LSF CS+ and CS− started to exhibit a significant disparity in the normalized SCR after the first 4 trials, but this disparity quickly vanished once the US was removed during the extinction stage (Fig. 1E; additional results for individual trial pairs of normalized SCR differences are available in the Supplementary Materials). All these findings indicated that the unconscious fear conditioning for emotionally neutral stimuli with LSF components was rapidly acquired but short-lasting. In Experiment 1b, we found no noticeable difference in the normalized SCR between the HSF CS+ and CS− in either the acquisition stage (*t*(27) =1.10, *P* = 0.447, Cohen’s *d* = 0.21) or the extinction stage (*t*(27) = 0.48, *P* = 0.635, Cohen’s *d* = 0.09) (Fig. 1D, right). Moreover, as illustrated in Fig. 1F, the normalized SCR difference had little change when we analyzed it by accumulating across trials in the 2 stages as in Experiment 1a.

The findings from Experiment 1 cannot be accounted for by participants’ implicit knowledge of the contingencies between the CS and US, as their shock expectancy for the CS+ did not significantly differ from the CS− in both the acquisition (Experiment 1a: CS+ vs. CS−: 2.16 vs. 2.13, *t*(27) = 0.38, *P* = 0.874, Cohen’s *d* = 0.07; Experiment 1b: CS+ vs. CS−: 2.15 vs. 2.14, *t*(27) = 0.16, *P* = 0.874, Cohen’s *d* = 0.03) and the extinction stages (Experiment 1a: CS+ vs. CS−: 2.32 vs. 2.25, *t*(27) = 0.83, *P* = 0.834, Cohen’s *d* = 0.16; Experiment 1b: CS+ vs. CS−: 2.21 vs. 2.15, *t*(27) = 1.426, *P* = 0.660, Cohen’s *d* = 0.27).

### Emotionally neutral stimuli with LSF and HSF elicit comparable SCRs during conscious fear conditioning

Experiment 2 aimed to examine whether the dependence of LSF information in the process of fear conditioning is only a prerequisite for the unconscious situation. In other words, when the CS+ and CS− were consciously perceived, we expected that both the LSF and the HSF information could be conditioned with fear. In Experiment 2a, all the CSs were visible and with LSF, while in Experiment 2b, all the CSs were visible and with HSF. All the other procedures were identical to that of Experiment 1. As shown in Fig. 1G (left), the results clearly demonstrated that the normalized SCR difference was significantly greater than zero in the acquisition stage of Experiment 2a (*t*(19) = 2.23, *P* = 0.038, Cohen’s *d* = 0.50) and 2b (*t*(18) = 3.36, *P* = 0.006, Cohen’s *d* = 0.77), indicating that the SCR induced by CS+ was greater than the SCR induced by CS− in the conscious fear conditioning, irrespective of whether the CSs were with LSF or with HSF. In addition to the independence of spatial frequency, there are 2 more important facets that the conscious fear conditioning was found to differ from the unconscious fear conditioning (see Fig. 1H and I). Firstly, the cumulative analysis showed that the normalized SCR difference between the CS+ and CS− did not reach significance until the last few trials in the acquisition stage, suggesting that the acquisition of fear conditioning for visible CS+ was not as fast as that for invisible CS+. Secondly, we found the normalized SCR difference remained significant even after removal of the US (i.e., in the extinction stage Fig. 1G, right, Experiment 2a: *t*(19) = 2.50, *P* = 0.029, Cohen’s *d* = 0.56; Experiment 2b: *t*(18) = 3.73, *P* = 0.006, Cohen’s *d* = 0.86).

Experiment 2 showed that when the participants were aware of the contingency of the CS and US, their shock expectancy was 2.40/1.76 in the acquisition stage and 2.40/1.60 in the extinction stage for the CS+/CS− with LSF, and 2.53/1.55 in the acquisition stage and 2.52/1.30 in the extinction stage for the CS+/CS− with HSF. The expectancy was significantly greater for the CS+ than the CS− irrespective of conditioning stage and spatial frequency (*ps* ≤ 0.001). Overall, the results of Experiments 1 and 2 on one hand showed that the unconscious fear conditioning can be characterized as an LSF-dependent, rapidly acquired process and on the other hand revealed that the conscious fear conditioning is independent of spatial frequency information and was slowly learned.

### Emotionally neutral stimuli with LSF but not HSF induce pupil dilation during unconscious fear conditioning

Experiment 3 further verified and characterized the unconscious fear conditioning using another classical measurement in fear conditioning research, i.e., the pupil diameter [25,26]. We extended the presentation duration of the CSs (from 2 s in Experiments 1 and 2 to 4 s in Experiment 3; see Fig. 2A) so that we could examine the dynamic change of pupil diameter duringfear conditioning without removing the CS+US trials. More importantly, we conducted more trials than the SCR experiments and further explore whether the rapid decline of unconscious fear response illustrated by the SCR results in the extinction stage was due to extinction or other possibilities, such as a quick habituation.

To further ensure the invisibility of the CSs for each participant, we introduced 2 orientation discrimination tasks, one before and the other after the main experiment. All the included participants in Experiment 3 performed at chance level in these 2 orientation discrimination tasks (Experiment 3a - before: 0.53 ± 0.08, t(16) = 1.67, P = 0.115, Cohen’s d = 0.40; after: 0.53 ± 0.07, t(16) = 1.55, P = 0.140, Cohen’s d = 0.38; Experiment 3b - before: 0.52 ± 0.07, t(16) = 1.22, P = 0.238, Cohen’s d = 0.30; after: 0.52 ± 0.08, t(16) = 1.02, P = 0.321, Cohen’s d = 0.25).

In Experiment 3a where the invisible CSs were presented with LSF, no significant differences were found when we compared the average pupil diameter across all trials recorded from the CS+ and the CS− trials (*P* > 0.05, see the rightmost panel in Fig. 2B). However, based on the rapidly appeared SCR found in Experiment 1, we illustrated the temporal profile of the pupil diameter difference between the CS+ and CS− by cumulating them at a pace of 8 trials, which had been proved to elicit noticeable SCR differences. As shown in Fig. 2B, the pupil diameter difference between the CS+ and CS− reached significance in the beginning 8 trials from 1.41 to 2.49 s after stimulus onset (*P* < 0.05, cluster corrected, see the leftmost panel in Fig. 2B). However, with more trials being accumulated, these responses seemed to gradually decline even in the acquisition phase (results for consecutive trial bins of the pupil diameter differences are also provided in the Supplementary Materials). In contrast to Experiment 3a, we did not find any significant results for CSs with HSF in Experiment 3b using the same analysis (Fig. 2C). Therefore, these results of Experiments 3a and 3b convergingly support that the unconscious fear conditioning can rapidly take place with LSF, emotionally neutral stimuli, even though it also quickly dissipates possibly due to habituation.

## Discussion

Learned fear serves to facilitate defensive behaviors when a threat approaches, thus increasing the organism’s chance of survival. The fear association could be effortlessly learned even when the fear-relevant stimuli are not perceived consciously [4,7,8]. The current study extends this notion and further demonstrates that such unconscious fear association can be acquired even for emotionally neutral stimuli, leading to stronger SCR and larger pupil size for CS+ versus CS−. More importantly, the unconscious fear conditioning ﻿ seems to rely more on the LSF information than the conscious fear conditioning. This observation is in accordance with the existence of a subcortical pathway tuned to processing the coarse, threat-related signals even without awareness.

In exploring the unconscious fear conditioning, most studies have selectively picked the fear-relevant stimuli as CSs [6–8] since the prevalence of the “prepared” theory. The current study, together with several studies employing fear-irrelevant stimuli as CSs [11–13,27], demonstrated that the fearful components of stimuli might not be necessary for the unconscious acquisition of conditioned fear, thus challenging the dominant position of the “prepared” theory in unconscious fear conditioning [9,10]. It is worth noting that the current study improved the masking technique (for some criticisms and suggested improvements in masking, see [28]) and conditioning procedures relative to previous ones. First, the design of Lipp et al. [27] did not strictly meet the requirements of unconscious operations (i.e., the unmasked blocks always preceded masked blocks), which may lead to a carryover effect of conscious fear learning to the process of unconscious fear acquisition. In the current study, the conscious and unconscious fear conditioning were in separate experiments, which maximally avoided the potential influence. More critically, the CFF technique we employed, is able to block the engagement of high-order cortical areas beyond early visual areas [21,22], guaranteeing effective and prolonged suppression of awareness compared with other masking techniques. Second, in studies of Balderston and his colleagues [12,13], a special 2-phase conditioning paradigm was used to measure the influence of masked fear conditioning on subsequent learning of unmasked CSs. This paradigm only indirectly reflected the associative fear responses to the masked fear-irrelevant stimuli. The current study adopted a classical Pavlovian conditioning procedure to reveal the direct relationship between the conditioned fear response and unconscious fear-irrelevant CSs [29]. The convergent results can thus be regarded as compelling evidence by far in support of the ability of emotionally neutral stimuli to elicit the unconscious fear conditioning.

Some researchers proposed a delay conditioning protocol (i.e., the US onset is delayed to the CS+ onset) rather than a trace conditioning (i.e., the US and CS+ are temporally separated; for more comparison between delay and trace conditioning, please refer to the review [24]) may be optimal for unconscious fear conditioning to be established with emotionally neutral stimuli, and the failure of earlier studies on this issue [3,4] can probably be attributed to this protocol differences too [12]. However, a follow-up study adopting the trace conditioning did also find unconscious fear conditioning with the neutral stimuli as the CSs [13]; on the other hand, though the current study employed the delay conditioning, unconscious fear conditioning was not observed for all emotionally neutral CSs but was spatial frequency dependent. Therefore, it is very likely that the spatial frequency of the CS rather than the specific conditioning protocol can explain why some emotionally neutral CSs can be associated with unconscious fear while others cannot.

Spatial frequency is one of the important characteristics of visual stimuli. It has been repeatedly observed that the behavioral and neural emotional responses were selectively elicited by LSF rather than HSF information of threat-related stimuli [15–18,20]. The current findings not only led support to the LSF selectivity in emotional responses [15–17] but also generalized to emotional processing in the absence of awareness [18,20]. These findings are also consistent with the postulation that there exists a threat-related and rapid subcortical, predominantly magnocellular [30,31] pathway that transmitted coarse information (i.e., LSF) to the amygdala bypassing the striate cortex [5,7,32–36]. Although some controversial arguments have been proposed [14,19,35,37–39], recent studies in nonhuman primates and advanced computational modeling of neuroimaging data provided relatively robust evidence for the existence and function of this pathway [19,40–45]. Furthermore, this subcortical pathway may be more important for unconscious fear processing, among other routes capable of transmitting conscious fear-relevant signals to the amygdala and thus probably independent of specific spatial frequency (e.g., McFadyen et al. [19] and our conscious fear conditioning results). By rendering the CSs invisible, the engagement of high-order cortical regions in visual processing was effectively blocked, leading the fear-relevant routes that transmit visible fear signals to the amygdala unable to work proficiently. From this view, our finding that the unconscious fear conditioning prioritized LSF information may be well explained by the reliance on the subcortical pathway tuned to LSF [15–17,35,37].

On the other hand, when the participants could consciously perceive the CS, the fear association can be formed regardless of whether the CS was with LSF or HSF. This result actually echoed with the aforementioned results of Schultz and Helmstetter [23] that the conscious fear conditioning can be established for both LSF and HSF as long as participants can perceive the features of the CSs. Since the amygdala also connects with cortical regions in the ventral visual stream that receives visual inputs predominantly from the parvocellular pathway tuned to HSF [16,35], it is conceivable that multiple fear-related routes, either cortical or subcortical, either specific or unspecific to spatial frequency [38,46], may play differential roles in the conscious and the unconscious fear processing.

Rather than simply different in the strength of neural activity, ﻿the conscious and the unconscious fear processing is found to be separated at a relatively early processing stage and evoke differential patterns of neural activity across brain regions [35]. A previous study found that although the overall magnitude of the unconscious fear learning was comparable to the conscious fear learning, they evolved differently over time [8]. Consistently, the present study also found that the unconscious fear conditioning can be rapidly acquired, even in a few trials, and faded out quickly after the removal of the US, whereas the conscious fear conditioning is relatively slowly learned and showed a retention of fear (Figs. 1 and 2). ﻿These results may demonstrate a complementary ﻿mechanism ﻿underlying conscious and unconscious fear conditioning: awareness is indispensable for the consolidation and expression of long-term memory but is not so for the initial alerting or visceral response that facilitates a stimulus to be associated with threat [47,48]. Considerable studies have revealed that the amygdala networks support the acquisition and expression of conditioned defensive behaviors [49–51]. However, due to strong habituation of amygdala response to repeated stimuli (e.g., identical CS+ with the US in several trials, see [52,53]), a conscious state is necessary to build and maintain a stable fear association; otherwise, it would lead to a rapid habituation as observed in the fear conditioning without awareness.

Lastly, 2 limitations of the study need to be mentioned here. One is that in the SCR experiments, the extinction stage immediately followed the acquisition stage during fear conditioning, which made it impossible to clearly delineate the time course of the extinction process. It awaits to be investigated by future studies where a delayed extinction session or an extinction retrieval test on the following day is conducted. Another limitation is the sample size. Although the sample size in our experiments of the unconscious fear conditioning was determined by the power analysis (see Materials and Methods), the sample size is still on the small side. However, as the SCR and pupil data converged at the same conclusion, our findings were highly consistent across experiments. Given the age-old debate on the role of awareness in classical fear learning, studies should seek to enlarge their sample size when replicating these effects in the future.

In summary, by measuring 2 classical physiological indices, the SCR and the pupillary response, the present study provides robust evidence that emotionally neutral stimuli can be efficiently associated with a negative outcome unconsciously. The findings further support that the unconscious fear processing relies more on the LSF information, which works in parallel with the conscious fear processing without apparent preference for spatial frequency. Accordingly, it is recommended for future studies to consider the spatial frequency of emotionally neutral stimuli when exploring the unconscious fear conditioning. Additionally, the present study is the first to show that pupillary response, as much sensitive as the SCR, can be used as an effective physiological readout of human unconscious fear conditioning and may be applied in future studies with ease. ﻿Moreover, the mechanisms underlying the unconscious fear learning may also facilitate the deepened understanding of the anxiety disorders or phobias.

## Materials and Methods

### Participants

A sample size of 16 participants would be sufficient (power = 0.80, α = 0.05) to detect a moderate effect in the unconscious fear conditioning (*d* = 0.77), according to a study using a similar design to the present study [8]. The inclusion criteria for eligible participants in Experiment 1 were as follows (see Preprocessing and statistical analysis for more details): (a) They could not consciously tell apart the orientations of the chromatic gratings under the CFF paradigm. (b) They showed reliable SCR. There were 56 participants in Experiment 1, with half of them for Experiment 1a (11 males; mean age = 21.71, *SD* = 2.71 y) and the other half for Experiment 1b (12 males; mean age = 22.39, *SD* = 3.22 y). Given that Experiment 2 aimed to examine the fear conditioning process at the conscious level, the inclusion criteria only required participants to have reliable SCR. There were 20 participants in Experiment 2a (11 males; mean age = 23.25, *SD* = 4.64 y) and 19 participants in Experiment 2b (8 males; mean age = 23.21, *SD* = 2.26 y). In Experiment 3, only participants who passed the awareness check task were included (see Preprocessing and statistical analysis for details). There were a total of 34 participants, 17 of whom participated in Experiment 3a (7 males, mean age = 22, *SD* = 1.62 y) and 17 of whom participated in Experiment 3b (6 males, mean age = 23.76, *SD* = 2.11 y). Participants were all naïve to the purpose of this study and with normal or correct-to-normal vision. Informed consent was obtained from all individual participants included in the study. All procedures performed in the study involving human participants were in accordance with the ethical standards of the institutional review board of the Institute of Psychology, Chinese Academy of Sciences (H17028) and with the 1964 Helsinki declaration and its later amendments or comparable ethical standards.

### Stimuli and apparatus

The visual stimuli were generated by MATLAB (The MathWorks, Natick, MA) together with the Psychophysics Toolbox extensions [54] and displayed on a liquid crystal display monitor (refresh rate: 60 Hz; resolution: 1,920 × 1,080 pixels). Participants rested their heads on a chin rest at a distance of 60 cm from the monitor. In all experiments, the CSs were chromatic gratings (45° clockwise or 45° counterclockwise from vertical) with a visual angle of 5°. The CSs with LSF were 1 cycle per degree (Experiments 1a, 2a, and 3a), while the CSs with HSF were 9 cycles per degree (Experiments 1b, 2b, and 3b). During the main experiment, all the CSs were presented against a background of random color noise (15° × 15°) to eliminate the possible visible artifacts (see Fig. 1A and B). In Experiments 1 and 3, the gratings were rendered invisible through rapidly counterphase flickering at a frequency of 30 Hz so that their orientations were completely smeared and only a pure yellow disk without any orientation information could be perceived (Fig. 1C, the top row). By contrast, in Experiment 2, the chromatic gratings (i.e., the CSs) were rendered visible by flickering at the same phase rather than counterphase (Fig. 1C, the bottom row). The US was a brief electric shock that was delivered from a transcutaneous current stimulator (STM200) through 2 surface electrocardiography electrodes positioned on the right forearm (near the wrist). In Experiments 1 and 2, we collected participants’ skin conductance data through 2 Ag-AgCl electrodes filled with electrolyte gel that were attached to the distal phalanges of the second and third fingers of the left hand (Biopac model TSD203 and GSR100C). The data were recorded at a sample rate of 1,000 Hz by AcqKnowledge software (Version 4.1). In Experiment 3, participants’ pupil diameter and 2-dimensional eye position of their left eye were measured with a video-based eye-tracking system (SMI, Berlin, Germany; sampling rate: 500 Hz). A standard 9-point calibration procedure was conducted to locate the gaze position on the screen. To maintain an accurate measure of pupil size, the observers were required to keep their eyes on the fixation cross and to refrain from blinking throughout the presentation of the CSs.

### Procedure and design

#### Experiment 1

Prior to the main experiment, the intensity of the US was determined individually through a scale from 0 (no sensation) to 5 (painful and intolerable). For each participant, the shock intensity subjectively rated as painful but tolerable (level 4) was adopted as the US intensity. The main experiment consisted of 2 successive stages: an acquisition stage and an extinction stage. In the acquisition stage, one orientation of the chromatic gratings (CS+, e.g., 45° clockwise from vertical) was paired with electric shocks on 50% of occasions (CS+US). Given the slow response properties of the SCR, this partial reinforcement protocol allowed us to avoid the overlap of the SCR induced by the US through only analyzing the CS+ trials without electric shocks. By contrast, the chromatic gratings with an orthogonal orientation (CS−, e.g., 45° counterclockwise from vertical) were never paired with shocks. There were a total of 24 trials in the acquisition stage: 8 CS+, 8 CS+US, and 8 CS−. In each trial, the invisible chromatic gratings (CS+US, CS+, or CS−) were displayed at the screen center for 2 s, with an electric shock delivered and coterminated with the CS+ for the last 150 ms in the CS+US trials (i.e., a delay conditioning). Then, after an 8-s blank, participants needed to answer 3 questions without a time limit by pressing correspondent buttons on the keyboard using their right hands (Fig. 1A). First, they should discriminate the orientation of the invisible grating in a 2-alternative forced-choice task (clockwise or counterclockwise from vertical). Second, they needed to rate their judgment confidence about their orientation discrimination (1-guess, 2-medium, and 3-sure). Third, they were required to report whether they expected that a shock would be delivered when the current trial started (i.e., a shock expectancy question with 1-expect no shocks, 2-uncertain, and 3-expect a shock). The former 2 questions were designed to objectively measure the effectiveness of CFF to suppress the CSs for each participant (see below analysis), while the third question was designed to assess participants’ awareness of the CS–US contingencies [55]. After the 3 questions were finished, there was an 8-sec intertrial interval. The extinction stage had 16 trials, including 8 CS+ trials and 8 CS− trials. Each trial followed the same procedure as that in the acquisition stage. During the extinction stage, the CS+ was presented without being paired with the US. It should be noted that there was no apparent interruption between the acquisition and extinction stages, thus participants were unable to be aware of which stage they were in. The orientation of the CS+ was counterbalanced across participants in the main experiment. The trial order was pseudo-randomized, with no more than 2 CSs of the same orientation being consecutively presented and the first and last trials in the acquisition stage always being the CS+US trial [24].

#### Experiment 2

Experiment 2 followed the same procedure and design as Experiment 1, except that participants were only required to complete the shock expectancy task (Fig. 1B).

#### Experiment 3

Different from the previous 2 experiments, a 2-alternative forced-choice, awareness check task was introduced to check whether the CSs were effectively fused into invisibility before and after the main experiment. In this task, participants were asked to discriminate the orientation of the chromatic gratings (rendered invisible using CFF as mentioned in Experiment 1) by pressing the left or right arrow key. Each task consisted of 30 trials.

The intensity of the electric shock was determined in a similar way in Experiment 1. As a finer scoring of the scale would produce a more accurate measurement, here we adopted another commonly used criterion in US calibration [24]. Participants subjectively rated the negative valence of the electric shock (i.e., the US) on a scale from 0 (not unpleasant at all) to 10 (extremely unpleasant). The shock intensity subjectively rated as “painful but tolerable” and with the negative valence higher than 7 was adopted as the US’s intensity in the main experiment.

As there was little evidence of the unconscious fear extinction in Experiment 1 (see Results), Experiment 3 only included the conditioning stage. In this stage, the CS+ was always paired with an electric shock in 100% of the trials (US). In each trial, the presentation duration of both the CS+ and the CS− was extended from 2 s (Experiments 1 and 2) to 4 s in Experiment 3. A US was delivered 250 ms ahead of the termination of the CS+ and co-ended with the CS+ (see Fig. 2A for an exemplar trial). Given that the pupil diameter responds relatively faster than the SCR and the presentation duration was extended to 4 s, we could avoid the confounding effect caused by the electric shocks in the CS+US trials by only focusing on the pupil diameter in the first 3 s after CS onset (see Preprocessing and statistical analysis below for details).

At the end of each trial, participants needed to answer 1 or 2 questions depending on their response to the first question. The first question was “Could you see a grating in the yellow disk? Yes or no”, for which participants reported by pressing the corresponding keys. If participants answered “Yes”, they should continue to answer the second question. The second question was “What is the orientation of the grating? Rightwards, leftwards, or unsure”, for which participants need to distinguish the orientation by pressing predefined buttons. These questions served as an additional awareness check during the main experiment, and for each participant, any trials in which the grating could be seen with its orientation correctly identified were further excluded from the analysis. After responses to the questions, there was a jittered intertrial interval of 3 to 5 s. During the entire experiment, participants were required to fix on a black cross in the middle of the screen, to minimize eye movements. There were 40 CS+ and 40 CS− trials, presented in a pseudo-randomized order. As in Experiments 1 and 2, the first trial was always reinforced and no more than 2 of the same trial type ever occurred consecutively.

### Preprocessing and statistical analysis

#### Experiment 1

First, we carried out several analyses to screen out participants who were not completely unaware of the orientations of the CSs. We only included participants whose accuracy of the orientation discrimination task was at chance level (according to a binomial test) and had no more than 8 correctly identified trials with confidence at 2 or 3. For those participants who met the above criteria, we also discarded their correct trials scored 2 or 3 on the confidence check [8]. On average, 0.02 ± 1.74 trials were excluded per participant in Experiment 1a, and 0.05 ± 2.13 trials were excluded per participant in Experiment 1b.

The raw skin conductance data from their unreinforced trials (i.e., the CS+ trials) were preprocessed using the Ledalab toolbox [56]. This toolbox could separate the event-related SCR from the slowly varying tonic activity of the raw skin conductance data through deconvolution. Firstly, the raw skin conductance data were down-sampling to 10 Hz. Secondly, the maximum value from 1 to 5.5 s after CS onset was defined as the SCR for each trial, and the SCR less than 0.02 μS was considered unreliable and converted to 0 following other studies [8,57]. Participants who had more than 8 trials of unreliable SCR were further excluded from the analysis. Thirdly, for each participant, all the SCR was normalized by dividing his/her largest SCR and square-root transformed to reduce skewness [58]. Lastly, the difference between the mean SCR triggered by the CS+ and CS− was also individually normalized by dividing their sum, $\frac{\mathrm{CS}+-\mathrm{CS}-}{\mathrm{CS}++\mathrm{CS}-}$ [8]. The normalized SCR difference was then submitted to a 1-sample *t* test separately for the acquisition stage and extinction stage. If the normalized SCR difference significantly deviates from zero, it is deemed as a sign of successful unconscious fear conditioning. To explore the stability of the normalized SCR difference over time, or how many trials were required to substantiate the observed conditioned response, we adopted a cumulative analysis similar to Raio et al. [8]. Specifically, the normalized SCR difference was gradually accumulated at a pace of 2 trials for the acquisition and extinction phases, respectively (i.e., acquisition: 1–2, 1–4, 1–6, 1–8; extinction: 9–10, 9–12, 9–14, 9–16), and evaluated against zero by the same 1-sample *t* tests. Finally, we examined whether participants would correctly anticipate an incoming shock even if they could not consciously see the orientation of the CSs. Their subjective expectancy scores were compared between the CS+ and CS− trials using paired-sample *t* tests. The multiple *t* tests conducted in the analysis were all false discovery rate corrected.

#### Experiment 2

The data analyses were almost the same as in Experiment 1. Here, 1 participant was excluded from the shock expectancy analysis due to a misunderstanding of the task instruction.

#### Experiment 3

To ensure that participants were unaware of the CSs, we performed 2 analyses. Firstly, we only included participants whose accuracy in the awareness check task before and after the main experiment was at chance level. Secondly, any trials that participants reported they saw the grating with its orientation correctly identified were excluded during the conditioning stage. This resulted in an average of 4.29 ± 3.5 trials per participant excluded in Experiment 3a and 2 ± 2.26 trials per participant excluded in Experiment 3b. Participants with over 20% of excluded trials were discarded from further analysis.

The raw pupil data were first preprocessed to remove artifacts. Firstly, blinks were replaced by linear interpolation over the missing data points [26,59]. To minimize the residual constriction after blinks, we set the interpolation window from 40 ms before the blinks to 80 ms after the blinks. Secondly, we filtered the entire pupil data using a 4-Hz low-pass filter to reduce the measurement noise. Thirdly, to control for between-subject variability, the entire pupil data (recorded in arbitrary units) were *z*-transformed for each participant, and all the following analyses were carried out with the pupil diameter expressed in *z*-scores. Fourthly, epochs from 0.5 s before to 3 s after the CS onset were extracted from the *z*-scored pupil diameter. The extracted pupil diameter was then baseline-corrected by subtracting the average pupil diameter across 0.5 s before the CS onset for each trial. Finally, trials with outliers exceeding 3.5 SDs were excluded.

After preprocessing, the normalized pupil diameter was submitted to a point-to-point paired *t* test to examine the difference between the CS+ and CS− conditions from 1 to 3 s after stimulus onset across all trials. The cumulative analysis was also performed on the normalized pupil difference over time, at a pace of 8 trials (i.e., 1–8, 1–16, 1–24, 1–32, and 1–40) for each participant, and the same paired *t* tests were performed on the pupil diameter from the CS+ and CS− trials for each accumulation group. The *P* values were corrected for multiple comparisons using a cluster-based permutation test which was performed by shuffling CS+/CS− labels for data epochs to create distributions under the null hypothesis. Paired *t* test was applied to the time curve derived from shuffled data. A *P* value of 0.05 was chosen to find out significant points. Clusters were then extracted and the largest summed *t* score of the clusters was passed to the null distribution. This procedure was repeated 1,000 times. The summed *t* score of the cluster derived from the unshuffled data epochs was compared to the 95th percentile of the null distribution, and those clusters with *t* scores larger than this threshold were labeled as significant clusters. All the abovementioned analyses were performed in MATLAB using custom scripts.

## Data Availability

All data and analysis codes used in this paper have been publicly available and can be obtained from [http://ir.psych.ac.cn/handle/311026/40100].

## Supplementary Materials

Fig. S1. Normalized SCR difference score for individual trial pairs across the 4 experiments. Fig. S2. Temporal profile of pupil diameter in fear conditioning with LSF or HSF stimuli.
