# Supplementary material for: Rapid Unconscious Acquisition of Conditioned Fear with Low-Spatial-Frequency but Emotionally Neutral Stimuli
Source: Research (Wash D C). 2023 Jun 27;6:0181. doi: 10.34133/research.0181 (PMC10298222; doi:10.34133/research.0181)
Supplement: Supplementary file 1 — Fig. S1. Normalized SCR difference score for individual trial pairs across the 4 experiments. Fig. S2. Temporal profile of pupil diameter in fear conditioning with LSF or HSF stimuli. [file research.0181.f1.docx]

**Supplementary Materials**

For the SCR data, we obtained the normalized SCR difference value on an individual trial pair basis (i.e., trials 1-2, trials 3-4, …, trials 15-16). The same one-sample t-tests were performed for each bin, and the significant bins were plotted (**Figure S1**). For the pupil data, we averaged the normalized pupil diameter at a pace of eight trials (i.e., 1–8, 9–16, 17–24, 25–32, and 33–40), and performed the paired t-tests as well. Same as the previous results, we found that the pupil diameter difference between the CS+ and CS- with LSF reached significance after onset in the beginning 8 trials (stage 1: t(16) = 2.52, p = .023; stage 2–5: ps > .05), but as the learning progresses, the significant pupil difference declined to zero (**Figure S2**).


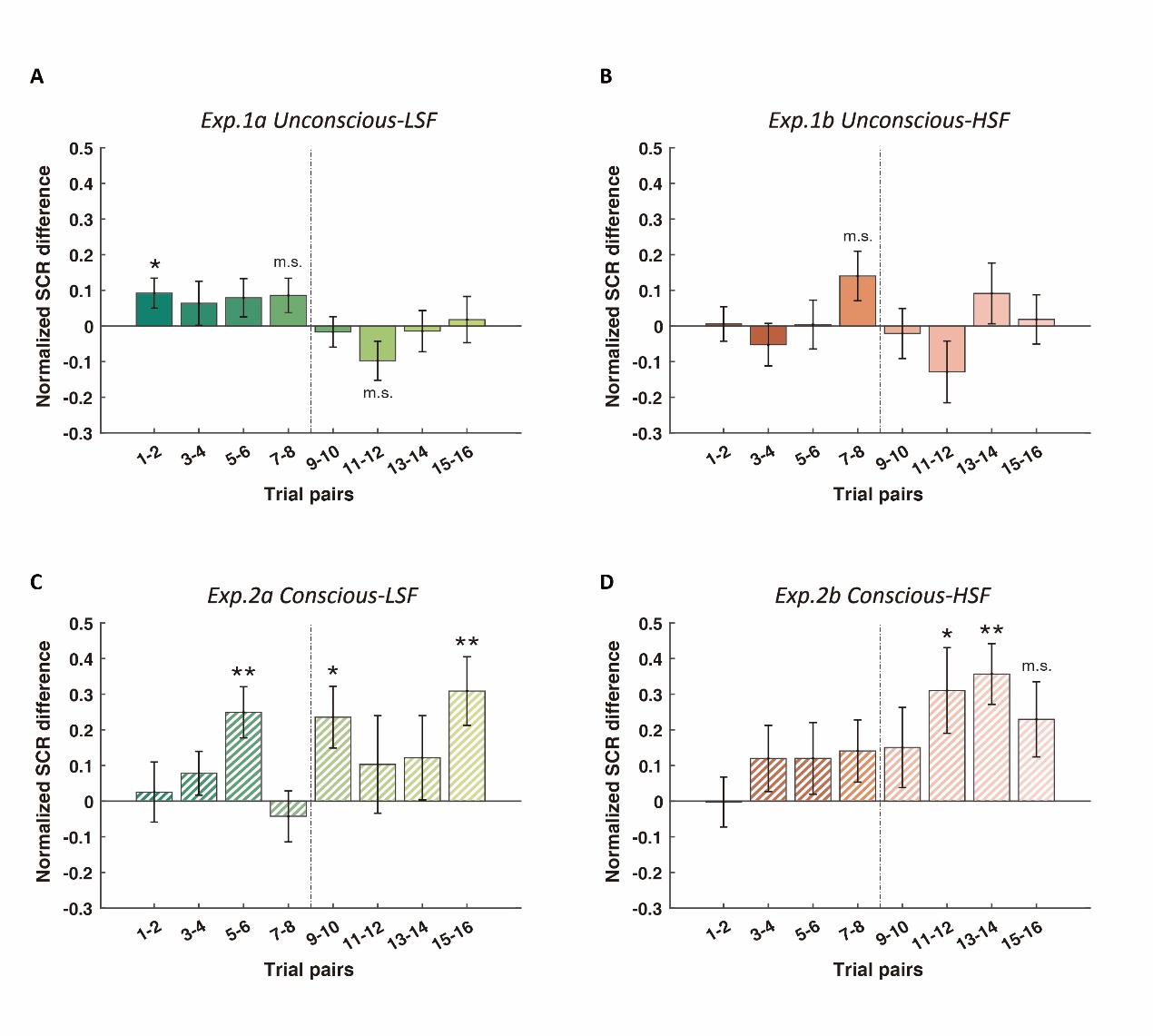


**Figure S1. Normalized SCR difference score for individual trial pairs across the four experiments.** (**A**) Experiment 1a: Unconscious-LSF. (**B**) Experiment 1b: Unconscious-HSF. (**C**) Experiments 2a: Conscious-LSF. (**D**) Experiments 2b: Conscious-HSF. Error bars indicate the standard errors of the mean.**p* < .05, ***p* < .01, m.s., marginally significant. All *p* values were uncorrected.


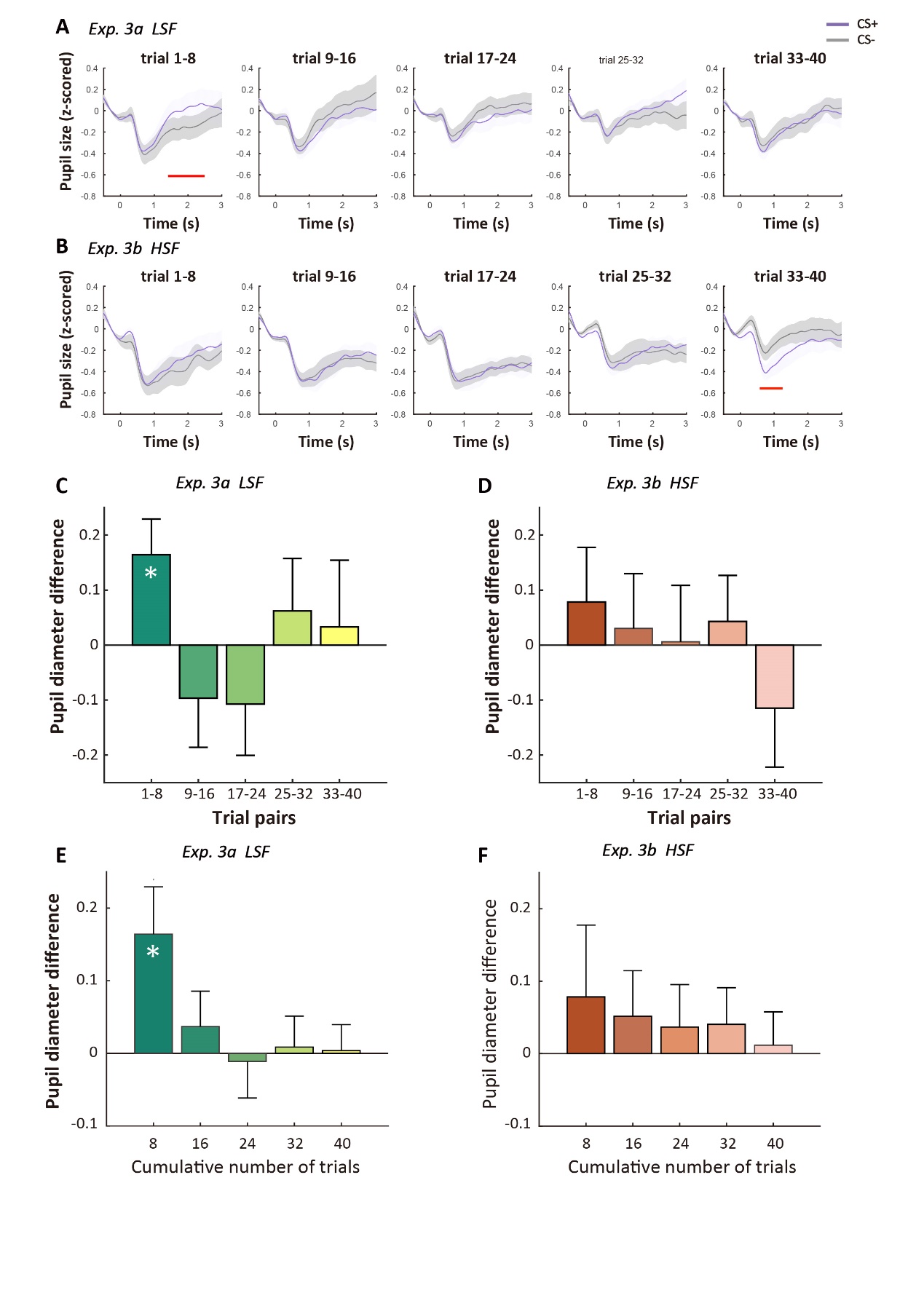


**Figure S2. Temporal profile of pupil diameter in fear conditioning with LSF or HSF stimuli.** (A and B) The averaged pupil diameter in response to the CS+ and CS- at a pace of eight trials in the LSF (A) and HSF (B) conditions, respectively. The red line denotes the time points when there were significant pupil diameter differences between the CS+ and CS- (corrected by cluster-based permutation). (C and D) The average pupil diameter differences between the CS+ and CS- with LSF (C) and HSF (D) in each group. Each bar represents the pupil diameter differences averaged across 1–8, 9–16, 17–24, 25–32, and 33–40 trials respectively. (E and F) The cumulative average pupil diameter differences between the CS+ and CS- with LSF (E) and HSF (F) in each accumulation group. Each bar represents the pupil diameter differences averaged across 1–8, 1–16, 1–24, 1–32, and 1–40 trials, respectively. Error bars indicate the standard errors of the mean. **p* < .05. All *p* values were uncorrected.
